# Prevalence and nature of childhood trauma among patients with schizophrenia and bipolar affective disorder

**DOI:** 10.1192/j.eurpsy.2023.2204

**Published:** 2023-07-19

**Authors:** A. M. Abdelkarim, T. Molokheya, N. A. Moubark

**Affiliations:** 1 Psychiatry Department, Alexandria University; 2 Psychiatry Department, Maamoura Psychiatric Hospital, Alexandria, Egypt

## Abstract

**Introduction:**

Epidemiological studies shows that exposure to early stress in the form of abuse and neglect in childhood increases the risk for later development of severe mental illneses such as schizophrenia and bipolar affective disorder.

**Objectives:**

•Study the prevalence of childhood trauma in a sample of adult patients diagnosed with schizophrenia and bipolar disorder.

•Identify potential differences of types of childhood trauma between both groups.

**Methods:**

This cross-sectional study was conducted on 200 patients admitted to the inpatient unit at Maamoura psychiatric hospital in Alexandria. Assessment of the history and nature of childhood trauma was done by Arabic version of childhood trauma questionnaire (CTQ).

**Results:**

There was no statistically significant difference between the two studied groups regarding past history of childhood trauma (p=0.397). (Table 1) Regarding the nature of childhood trauma, the history of emotional abuse and physical neglect was more commonly associated with developing bipolar disorder whereas the history of physical abuse was sihnficantly more commonamong schizophrenia patients. (Table 2)

**Conclusions:**

History of childhood trauma is common among adult patients with schizophrenia and bipolar disorder with no significant difference between both groups. Nature of trauma may be diffirent where physical abuse might be correlated with later development of schizophrenia.

**Disclosure of Interest:**

None Declared

